# Characterization of rare histological subtypes of ovarian cancer based on molecular profiling

**DOI:** 10.1002/cam4.4927

**Published:** 2022-06-08

**Authors:** Nobutaka Takahashi, Keiichi Hatakeyama, Takeshi Nagashima, Keiichi Ohshima, Kenichi Urakami, Ken Yamaguchi, Yasuyuki Hirashima

**Affiliations:** ^1^ Division of Gynecology Shizuoka Cancer Center Sunto‐gun Shizuoka Japan; ^2^ Medical Genetics Division Shizuoka Cancer Center Research Institute Sunto‐gun Shizuoka Japan; ^3^ Cancer Diagnostics Research Division Shizuoka Cancer Center Research Institute Sunto‐gun Shizuoka Japan; ^4^ SRL Inc. Shinjuku‐ku Tokyo Japan; ^5^ Shizuoka Cancer Center Sunto‐gun Shizuoka Japan

**Keywords:** clear cell carcinoma, molecular profiling, non‐epithelial tumor, ovarian tumor

## Abstract

**Objective:**

Pan‐cancer analysis across The Cancer Genome Atlas has revealed the molecular profiles of major types of carcinomas. High‐grade serous carcinomas (HGSCs) have been characterized; however, in ovarian cancer, the profile of carcinoma with minor histopathological changes remains unclear. This study aimed to perform the molecular profiling of rare malignant ovarian tumors, including non‐epithelial tumors (NETs; germ cell tumors and sex cord tumors) and clear cell carcinoma (CCC), to determine how they differ from the major HGSCs.

**Methods:**

Sixty‐nine malignant ovarian tumors surgically resected at the Shizuoka Cancer Center between January 2014 and March 2019 were classified based on their histopathological types. The germline and somatic mutations in these carcinomas, including NETs, were determined using next‐generation sequencing. Gene expression analysis was performed to investigate the major pathways of drug resistance, which is a characteristic of CCC.

**Results:**

NETs harbored copy‐neutral loss of heterozygosity, accompanied by a high homologous recombination deficiency score; germline mutations of *PALB2* and *BARD1* were identified in two patients with NET. In chemoresistant CCC, the epithelial‐mesenchymal transition pathway was activated regardless of ABC transporter expression.

**Conclusion:**

This study revealed some genomic characteristics of rare malignant ovarian tumors, including NETs and CCC.

## Graphical abstract

This study revealed molecular features of rare malignant ovarian tumors, including non‐epithelial tumors (NETs; germ cell tumors and sex cord tumors) and clear cell carcinoma (CCC). NETs showed high HRD scores similar to those of high‐grade serous carcinomas and germline mutations in BARD1 and PALB2 are related to HR and the accumulation of cnLOH. Chemoresistant CCC upregulated the expression of genes in the EMT pathway.

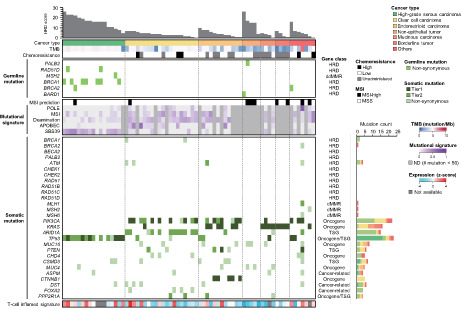

## 1 INTRODUCTION

Among all gynecological cancer types, advanced ovarian cancer has a poor prognosis, with more than 80% of cases recurring after standard treatment. Optimal debulking surgery and chemotherapy are the main treatments for advanced ovarian cancer.^1^, ^2^, ^3^ Although chemotherapy is particularly effective in treating serous cancers, most recur and are difficult to cure.^4^, ^5^ For two decades or more, platinum‐based chemotherapy has been the key regimen, with no other therapy being more effective.^6^, ^7^ With the advent of poly (ADP‐ribose) polymerase (PARP) inhibitors, ovarian cancer chemotherapy has undergone rapid changes in recent years; this class of drugs has been reported to be more effective in patients with germline or somatic mutations in *BRCA1/2* genes and/or with homologous recombination deficiency (HRD).^8^, ^9^, ^10^ The Cancer Genome Atlas (TCGA) project has revealed the distribution of patients with *BRCA1/2* mutations and/or HRD in ovarian serous carcinoma based on molecular profiling.^11^ In TCGA, homologous repair alteration was observed in 51% of the patients, including *BRCA1/2* gene abnormality (33%). Therefore, patients with ovarian serous carcinoma strongly benefit from treatment with PARP inhibitors.

Approximately 90% of ovarian malignancies are epithelial tumors, whereas several rare tumors, including germ cell tumors and sex cord tumors, are categorized as non‐epithelial tumors (NETs).^12^ Recently, mutational analysis has characterized germ cell tumors as harboring a low mutation rate along with amplified *PIK3CA*.^13^ However, in NETs, not much is known about germline mutations in *BRCA1/2* and HRD that affects the efficacy of PARP inhibitors.

In Europe and the United States, clear cell carcinoma (CCC) is a rare tumor accounting for approximately 10% of all ovarian cancers.^14^, ^15^ In Japan, 20%–25% of ovarian cancers are categorized as CCC, which constitutes one of the major histological types.^15^, ^16^ CCC is more resistant to chemotherapy than other histological types of ovarian malignancies. Mutation analysis using next‐generation sequencing has been performed for CCC, and its molecular profile has been clarified.^17^, ^18^ However, the pathways involved in drug resistance in CCC remain unclear.

This study focused on this rare cancer of the ovary; mutation and gene expression analyses were performed in Japanese patients with ovarian cancer using next‐generation sequencing. The profiles of epithelial carcinomas, including ovarian serous carcinomas and CCCs, and NETs, which are rare, were compared for chemoresistance, *BRCA1/2* mutations, and HRD, which affect the efficacy of PARP inhibitors. The aim of our analysis was to provide a new perspective for treating rare histological types of ovarian cancer other than ovarian serous carcinoma.

## 2 MATERIALS AND METHODS

### 2.1 Ethical statement

Study approval was obtained from the Institutional Review Board of the Shizuoka Cancer Center (authorization number 25‐33). All patients enrolled in the study provided written informed consent. Experiments using clinical specimens were conducted following the approved Japanese ethical guidelines (Human Genome/Gene Analysis Research, 2017, published by the Ministry of Health, Labor, and Welfare; https://www.mhlw.go.jp/stf/seisakunitsuite/bunya/hokabunya/kenkyujigyou/i‐kenkyu/index.html).

### 2.2 Clinical samples

Ovarian tumor samples were selected from our previously established sample collection comprising 5521 tumor specimens.^19^ Tumor and adjacent tissues (≥0.1 g) were isolated from fresh specimens surgically collected at our center between January 2014 and March 2019 and were clinically assessed by a pathologist. More detailed in‐house procedures have been provided in a previous report.^19^ Clinical characteristics of the patients with malignant ovarian tumors, including age, histopathology, cancer stage, recurrence, and chemoresistance, were retrieved from their electronic medical records. Platinum‐containing chemotherapy is the standard initial chemotherapy for ovarian cancer,^4^, ^5^ and its efficacy is generally evaluated based on platinum sensitivity. We included only chemotherapy‐refractory patients whose best response to platinum‐based chemotherapy was stable disease or disease progression and who were classified as “chemoresistant.”

### 2.3 Datasets used for the analysis of somatic alterations

Fresh frozen sections were used for sequencing and microarray analysis. The data have been deposited to the National Bioscience Database Center (Research ID, hum0127, https://humandbs.biosciencedbc.jp/en/). From these datasets, we extracted the data required for molecular profiling analysis, including tumor mutation burden (TMB), copy number variation (CNV), mutational signature, and gene expression signature. Variants were classified into Tier 1 (pathogenic) or Tier 2 (likely pathogenic) according to a previous report.^19^ Mutational signature analysis was performed for all cases except those with less than 50 mutations using deconstructSigs^20^ or COSMIC mutational signature v3. (https://cancer.sanger.ac.uk/cosmic/signatures). Somatic CNVs were determined using saasCNV.^21^ The CNV size was determined by summing loss (CNV ≤1.5), gain (CNV ≥2.5), and copy‐neutral loss of heterozygosity (cnLOH) in the genome, based on estimates from whole‐exome sequencing (WES). Microsatellite instability (MSI) was predicted using MSIsensor, and MSI signatures were determined using reported algorithms.^19^, ^22^

### 2.4 Gene expression analysis

Total RNA was extracted from the samples using RNAlater solution (Thermo Fisher Scientific). The RNA was purified, amplified, and fluorescently labeled using the One‐Color Low Input Quick Amp Labeling Kit (Agilent Technologies). Fluorescently labeled cRNAs were hybridized to a SurePrint G3 Human Gene Expression 8 × 60K v2 Microarray (Agilent Technologies). Expression signature and pathway analyses were conducted based on gene expression in the tumor tissues. Gene expression data of the ABC transporter family were extracted from the microarray dataset. A T‐cell‐inflamed gene expression profile (GEP) score was determined by weighted summation of normalized expression values for 18 genes included in a previously reported tumor inflammation signature that were found to define tumor type‐independent features of the tumor microenvironment and to predict clinical outcomes for drugs targeting the programmed‐death (PD)‐1/PD ligand‐1 signaling pathway.^23^, ^24^, ^25^ Pathway analysis was conducted using gene set enrichment analysis (GSEA)^26^, ^27^ with the Hallmark gene set (v7.2) (https://www.gsea‐msigdb.org/gsea/msigdb/index.jsp).

### 2.5 Statistical analysis

One‐way analysis of variance followed by Welch's *t*‐tests was used to compare the expression signatures between the chemoresistant and non‐chemoresistant groups. The Benjamini–Hochberg method was used for controlling the false discovery rate in GSEA (*q* < 0.05). Differences were considered significant at *p* < 0.01. Statistical analyses were conducted in R v4.1.0.

## 3 RESULTS

### 3.1 Sample distribution of malignant ovarian tumors

**FIGURE 1:**
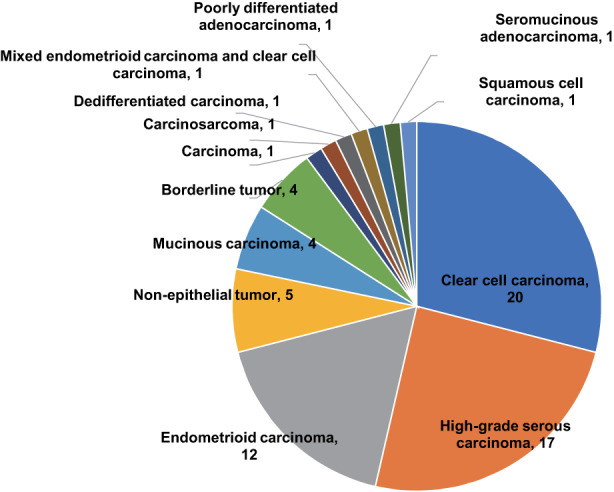
Histological distribution of 69 tumor samples of ovarian malignancies.

WES and targeted panel sequencing of patients with cancer were conducted in our previous study, Project HOPE.^28^ For the current study, 79 ovarian tumor samples with detailed clinical information were extracted from the HOPE cohort (comprising 5521 tumor specimens). As this study focused on malignant ovarian tumors, benign (*n* = 10) tumors were excluded. The malignant tumors (*n* = 69) included 20 CCCs, 17 high‐grade serous carcinomas (HGSCs), 12 endometrioid carcinomas (ECs), five NETs (immature teratoma, two; yolk sac tumor, one; mixed germ cell tumor, one; and juvenile granulosa cell tumor, one), four mucinous carcinomas, four borderline tumors (seromucinous, two; and mucinous, two), and seven others (Figure 1). The clinical characteristics of all patients with malignant tumors are shown in Table 1.

**TABLE 1:** Clinical characteristics of patients with malignant ovarian tumors

	CCC (*n* = 20)	HGSC (*n* = 17)	EC (*n* = 12)	NET (*n* = 5)	MC (*n* = 4)	Border (*n* = 4)	Others (*n* = 7)	All (*n* = 69)
Age (years, median [range])	58 (44–76)	65 (43–77)	57 (39–87)	34 (21–78)	68 (51–72)	61 (39–71)	67 (44–74)	59 (21–87)
Stage								
I	13	0	10	2	4	4	3	36
II	2	2	0	0	0	0	0	4
III	2	9	1	3	0	0	1	16
IV	3	6	1	0	0	0	3	13
Recurrence								
+	3	6	1	0	0	0	4	14
−	17	11	11	5	4	4	3	55
Chemoresistance								
+	3	0	1	0	0	0	4	8
−	17	17	11	5	4	4	3	61

Abbreviations: Border, borderline tumor; CCC, clear cell carcinoma; EC, endometrioid carcinoma; HGSC, high‐grade serous carcinoma; MC, mucinous carcinoma; NET, non‐epithelial tumor.

### 3.2 Mutational landscape of malignant ovarian tumors

**FIGURE 2:**
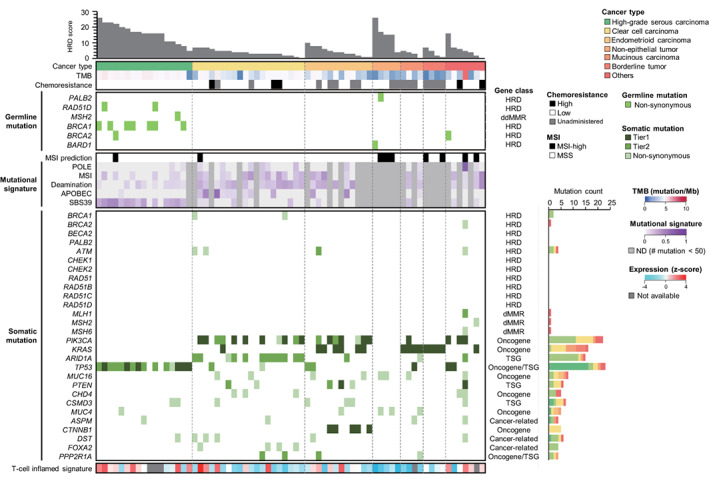
Mutation profiles of samples classified based on histopathology. Tier 1 and Tier 2 are classes of cancer‐associated mutations, as defined in a previous report.^19^ HRD, homologous recombination deficiency; MSI, microsatellite instability; TMB, tumor mutation burden.

To investigate the mutational landscape of malignant ovarian tumors in Japanese patients, WES, targeted panel sequencing, known fusion gene sequencing, and GEP were used. Figure 2 shows the mutation profiles of the samples classified based on histopathology. The HRD score of HGSCs was higher than that of other histopathological samples. *TP53* (88%, 15/17) mutation was identified in HGSC samples. *PIK3CA* (55%, 11/20) and *ARID1A* (60%, 12/20) mutations were identified in CCC. *PIK3CA* (58%, 7/12) and *KRAS* (50%, 6/12) mutations were identified in EC. These trends in somatic mutation accumulation were consistent with previously reported trends.^11^, ^29^, ^30^ The T‐cell‐inflamed GEP signature, predicting the effect of immune checkpoint inhibitors (ICIs), was highly expressed in ovarian carcinoma, especially in HGSC, although the TMB was <10 mutations/Mb (Figure S1)*. SBS39* has been recently identified^31^ in HGSC and was detected in 88% (15/17). In CCC, *FOXA2* has been identified (20%, 4/20).^18^ Interestingly, germline mutations in *PALB2* and *BARD1* and a high HRD score were identified in NETs, a rare tumor among malignant ovarian tumors.

**FIGURE 3:**
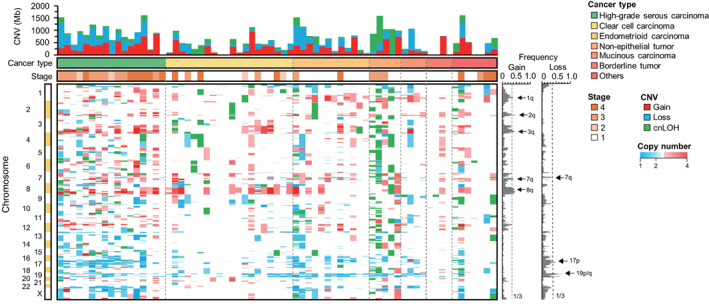
Profiling of copy number variation (CNV) in the histopathological groups. Order of samples as in Figure 1. In the upper right section, cancer stages are displayed in different colors. Black indicates the dead status of samples. Red indicates copy number gain. Blue indicates copy number loss. Green indicates copy‐neutral loss of heterozygosity (cnLOH). Arrows indicate chromosomal regions with CNVs >1/3.

Next, CNV analysis was conducted to investigate chromosomal aberration in malignant ovarian tumors (Figure 3). Amplification of 1q31.1‐1q31.3, 3q24‐3q26.33, and 8q12.1‐8q24.23 was observed in 36%–39%, 34%–46%, and 38%–46% of all samples, respectively. Amplification was detected in HGSCs (76%, 13/17), followed by CCCs (60%, 12/20), and ECs (58%, 7/12). cnLOH was detected in NETs (immature teratoma, two; and yolk sac tumor, one). No significant difference in gain and loss in other chromosomes was observed among the subgroups.

### 3.3 Pathway analysis of malignant ovarian tumors

Chemotherapy is one of the main treatment strategies for advanced malignant ovarian tumors. Chemotherapy is effective in treating most ovarian tumors, such as HGSCs, but not in some histological types. CCC is well known for its chemoresistance compared to other ovarian histological types. To characterize the biological pathways of CCCs involved in drug resistance, GSEA was performed between a chemoresistant group (*n* = 3) and a non‐chemoresistant group (*n* = 17) in CCC. In the former, gene sets associated with epithelial‐mesenchymal transition (EMT) were significantly enriched (Table 2). To confirm the variation in gene expression related to cytotoxic drug efflux, the gene expression levels of membrane proteins, including ABC transporters, were analyzed based on GEP. Although *HNF1B* and *ANXA4* were highly expressed in all CCCs, no significant differences in gene expression of the membrane proteins were observed between the chemoresistant and non‐chemoresistant groups (Figure S2).

**TABLE 2:** Gene set enrichment analysis of chemoresistant and non‐chemoresistant groups in CCC

Chemoresistant CCC	Gene set	NES	FDR *q*‐value
Upregulated (chemoresistant)	HALLMARK_EPITHELIAL_MESENCHYMAL_TRANSITION^a^	2.91	<0.001
	HALLMARK_TNFA_SIGNALING_VIA_NFKB^a^	2.74	<0.001
	HALLMARK_UV_RESPONSE_DN	2.24	<0.001
	HALLMARK_MYOGENESIS	2.16	<0.001
	HALLMARK_HYPOXIA^a^	2.14	<0.001
	HALLMARK_MITOTIC_SPINDLE	2.05	<0.001
	HALLMARK_INFLAMMATORY_RESPONSE	2.02	<0.001
	HALLMARK_IL2_STAT5_SIGNALING	1.97	<0.001
	HALLMARK_KRAS_SIGNALING_UP^a^	1.95	<0.001
	HALLMARK_ANGIOGENESIS^a^	1.95	<0.001
	HALLMARK_TGF_BETA_SIGNALING^a^	1.85	0.001
	HALLMARK_ANDROGEN_RESPONSE	1.83	0.001
	HALLMARK_APICAL_JUNCTION	1.82	0.001
	HALLMARK_APOPTOSIS	1.78	0.001
	HALLMARK_COAGULATION	1.67	0.004
	HALLMARK_HEDGEHOG_SIGNALING	1.65	0.004
	HALLMARK_UV_RESPONSE_UP	1.65	0.004
	HALLMARK_COMPLEMENT	1.61	0.005
Downregulated (non‐chemoresistant)	HALLMARK_INTERFERON_ALPHA_RESPONSE	−1.96	<0.001
	HALLMARK_INTERFERON_GAMMA_RESPONSE	−1.71	0.004

Abbreviations: CCC, clear cell carcinoma; FDR, false discovery rate; NES, normalized enrichment score.

^a^ Gene sets associated with epithelial‐mesenchymal transition.

## 4 DISCUSSION

Approximately 90% of ovarian malignancies are categorized as epithelial tumors, whereas in the remaining 10%, non‐epithelial cells are the predominant population.^12^ Germ cell tumors and sex cord‐stromal tumors, known as rare cancers, are largely composed of non‐epithelial components.^12^ Despite the differences in the histopathological types, these tumors were considered NETs in this study. The histogenesis and clinical features of NETs are different from those of epithelial tumors,^12^, ^32^ suggesting that NETs have different molecular features. The differences in histopathology may have influenced this analysis; therefore, future studies including a higher number of NET cases are warranted.

The HRD scores of these tumors were as high as those of HGSCs, against which PARP inhibitors are effective. Germline truncating mutations of *PALB2* (c.3350+5G>A, splice region) and *BARD1* (p.Q206fs) were detected in immature teratomas (classified as germ cell tumors), which showed particularly high HRD scores. BARD1 forms a complex with BRCA1 and is involved in recombinant DNA repair.^33^ PALB2 was identified as a protein that interacts with BRCA2 and functions in DNA repair by homologous recombination (HR).^34^ The above variants may lead to defective HR and subsequently, a high HRD score. NETs with high HRD scores do not harbor the *BRCA1* and *BRCA2* mutations seen in HGSC; however, they may still benefit from PARP inhibitors.

In the analysis of chromosomal CNV, cnLOH was detected in broad chromosomal regions in immature teratomas and yolk sac tumors (classified as germ cell tumors) with a high HRD score, whereas it did not accumulate in HGSC. cnLOH is associated with aberrations in DNA double‐strand break repair in patients with leukemia.^35^ Meiotic aberrations that can induce cnLOH have been reported in immature teratomas derived from the ovary.^36^ These reports imply a relationship between HRD and cnLOH; incomplete DNA repair by HR in germ cell tumors may preferentially lead to cnLOH, unlike the chromosomal instability seen in HGSCs.

TMB is used as a biomarker for predicting the response to ICIs in many cancer types.^37^ Some ovarian cancers respond to ICIs.^38^ Large‐scale analyses have indicated that the T‐cell‐inflamed GEP signature predicts responses to ICIs.^24^ HGSC highly expressed the T‐cell‐inflamed GEP signature independent of TMB; therefore, ICIs may be effective in treating HGSC.

In Japan, CCC accounts for 20%–25% of ovarian cancers and is one of the major histological types.^15^, ^16^ Although CCC is one of the most common chemoresistant cancers,^39^, ^40^ the mechanism underlying drug resistance in this cancer type remains unclear. Our comparative analysis of chemoresistant and nonresistant groups in CCC revealed that the expression of genes in the EMT‐related pathway was enhanced in the resistant group, regardless of the expression of the ABC transporter family. The activation of the EMT pathway confers drug resistance in lung cancer and pancreatic cancer.^41^, ^42^ Moreover, the high expression of genes in the EMT pathway in ovarian CCC is reportedly associated with poor prognosis.^43^ In our study, genes in the EMT pathway were highly expressed in chemotherapy‐refractory cases of ovarian CCC, suggesting that this pathway plays a central role in chemotherapy resistance.

Our analysis of a small number of rare ovarian cancers revealed new features of NETs and ovarian CCC. However, the number of analyzed samples were insufficient for a robust statistical analysis. To validate our characterization of rare ovarian cancers, further analysis of a large number of cases would be required in the future.

In this study, we focused our analysis on a rare histological type of ovarian cancer. Although germ cell tumors are NETs, they showed high HRD scores similar to those of HGSCs. We found that germline mutations in *BARD1* and *PALB2* are related to HR and the accumulation of cnLOH. We also found that the expression of genes in the EMT pathway was upregulated in CCC with chemotherapy resistance. Cross‐sectional molecular profiling of malignant ovarian tumors may allow the characterization of several such rare ovarian tumors in the future.

## AUTHOR CONTRIBUTIONS

Nobutaka Takahashi and Keiichi Hatakeyama contributed equally to this work and designed the study. Keiichi Hatakeyama, Takeshi Nagashima, Keiichi Ohshima, and Kenichi Urakami. performed the experiments. Keiichi Hatakeyama and Nobutaka Takahashi analyzed the data. Takeshi Nagashima and Keiichi Hatakeyama performed statistical analyses. Keiichi Hatakeyama and Nobutaka Takahashi wrote the paper, for contributions by Ken Yamaguchi and Yasuyuki Hirashima. All authors contributed to the approval of the final version of the manuscript.

## CONFLICT OF INTEREST

The authors have no conflict to disclose.

## ETHICAL APPROVAL STATEMENT

Study approval was obtained from the institutional review board of the Shizuoka Cancer Center (authorization number 25‐33). Experiments using clinical specimens were conducted following the approved Japanese ethical guidelines (Human Genome/Gene Analysis Research, 2017, provided by the Ministry of Health, Labor, and Welfare; https://www.mhlw.go.jp/stf/seisakunitsuite/bunya/hokabunya/kenkyujigyou/i‐kenkyu/index.html).

## PATIENT CONSENT STATEMENT

All patients enrolled in the study provided written informed consent.

## Supporting information


Figure S1

Figure S2

## Data Availability

The data that support the findings of this study are available from the corresponding author, upon reasonable request. Following the journal's guidelines, the data have been deposited to the National Bioscience Database Center Human Database as “Controlled‐Access Data” (Research ID, hum0127, https://humandbs.biosciencedbc.jp/en/).
