# Supplementary material for: Characterization of rare histological subtypes of ovarian cancer based on molecular profiling
Source: Cancer Med. 2022 Jun 8;12(1):387–95. doi: 10.1002/cam4.4927 (PMC9844652; doi:10.1002/cam4.4927)
Supplement: Supplementary file 1 — Figure S1 [file CAM4-12-387-s002.pdf]

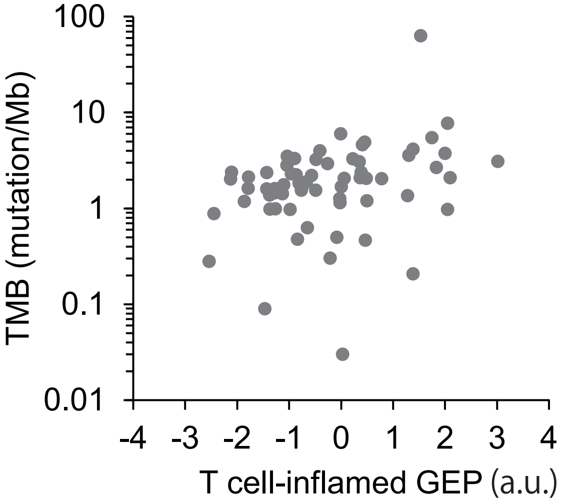

Figure S1. Relationship between T-cell inflamed gene expression profile and tumor mutational burden (TMB) in high-grade serous carcinoma.
