# Supplementary material for: Characterization of rare histological subtypes of ovarian cancer based on molecular profiling
Source: Cancer Med. 2022 Jun 8;12(1):387–95. doi: 10.1002/cam4.4927 (PMC9844652; doi:10.1002/cam4.4927)
Supplement: Supplementary file 2 — Figure S2 [file CAM4-12-387-s001.pdf]

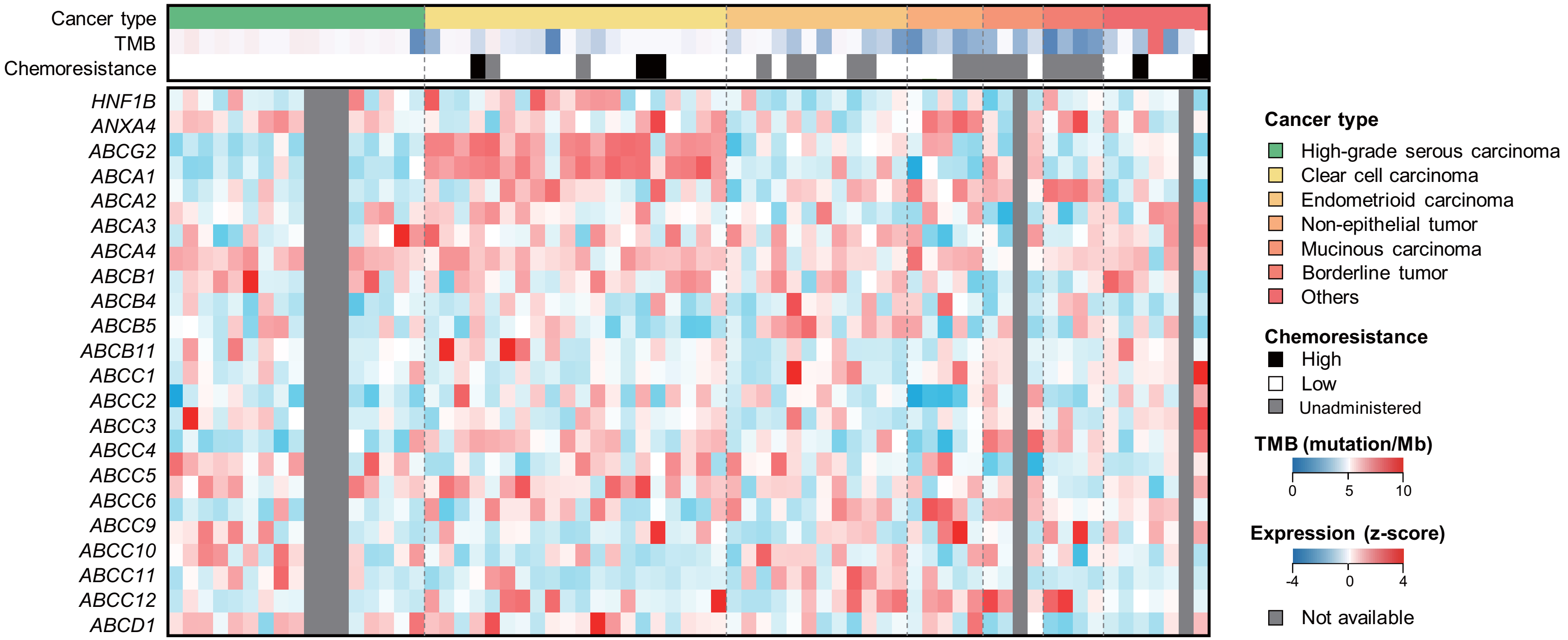

Figure S2. Expression of drug resistance-related genes in the histopathological groups. The order of the samples corresponds to that in Figure 1. Chemoresistant or microsatellite instability (MSI)-high samples are shown in black. Tumor mutational burden (TMB) > 5 mutations/Mb is indicated by a red gradient. Gene expression is represented by the z-score.
